# Dr. M. S. Buchanan

**Published:** 1860-09

**Authors:** 


The City of Glasgow has recently met with a bereavement in the loss of Dr. M. S. Buchanan, who was one of its most popular and successful surgeons and teachers. He graduated in Edinburgh, in 1818, and in 1829 was appointed Surgeon to the Royal Infirmary. Seven years subsequently he commenced to teach Anatomy in the Portland Street School, and in 1841 was made Professor of Anatomy in Anderson's University, a position which he held up to the time of his death. Dr. Buchanan was a most zealous and cultivated anatomist, as well as a popular clinical lecturer and good operator.

